# Antitumor activity of resveratrol against human osteosarcoma cells: a key role of Cx43 and Wnt/β-catenin signaling pathway

**DOI:** 10.18632/oncotarget.22810

**Published:** 2017-11-30

**Authors:** Da Xie, Gui-Zhou Zheng, Peng Xie, Qi-Hao Zhang, Fei-Xiang Lin, Bo Chang, Qin-Xiao Hu, Shi-Xin Du, Xue-Dong Li

**Affiliations:** ^1^ Department of Orthopedics, The Third Affiliated Hospital (The Affiliated Luohu Hospital) of Shenzhen University, Shenzhen 518000, Guangdong, P. R. China

**Keywords:** osteosarcoma, resveratrol, connexin 43, Wnt/β-catenin signaling, antitumor activity

## Abstract

Osteosarcoma is a high-grade bone sarcoma with strong invasive ability. However, treatment with traditional chemotherapeutic drugs is limited by low tolerability and side effects. Resveratrol has been reported previously to have selective antitumor effect on various tumor cells while little is known about its effects and underlying mechanism in osteosarcoma biology. In this study, we found that resveratrol inhibits proliferation and glycolysis, induces apoptosis and reduces the invasiveness of U2-OS cells *in vitro*. After treatment with resveratrol, the expression of related Wnt/β-catenin signaling pathway target genes, such as β-catenin, c-myc, cyclin D1, MMP-2 and MMP-9, was downregulated and an increased E-cadherin level was observed as well. Additionally, the dual luciferase assay results also indicated that resveratrol suppressed the activity of Wnt/β-catenin signaling pathway. Interestingly, we noticed that the expression of connexin 43 (Cx43) increased with the prolongation of resveratrol treatment time. To further investigate the relationship between Cx43 and the Wnt/β-catenin signaling pathway in osteosarcoma, we used lentiviral-mediated shRNA to knockdown the expression of Cx43. Knockdown of Cx43 activated the Wnt/β-catenin signaling pathway, promoted proliferation and invasion, and inhibited apoptosis of U2-OS cells. Taken together, our results demonstrate that the antitumor activity of resveratrol against U2-OS cells *in vitro* occurs through up-regulating Cx43 and E-cadherin, and suppressing the Wnt/β-catenin signaling pathway. Moreover, Cx43 expression is negatively related to the activity of the Wnt/β-catenin pathway in U2-OS cells.

## INTRODUCTION

Osteosarcoma is the most frequent primary malignant bone tumor derived from bone mesenchymal stem cells, and caused by genetic and epigenetic changes that block osteoblast differentiation and result in a high potential for local invasion and distant metastasis [1, 2]. It mostly occurs in regions of active bone growth, such as the knee joint, distal femur and upper tibia, with a peak incidence in children and adolescents [3]. Many genes, proteins and signaling pathway changes have been found in osteosarcoma, but the pathogenesis of osteosarcoma is quite complex and has not yet been clarified [4]. Currently, the main treatment regimen for osteosarcoma consists of three parts: preoperative chemotherapy, resection of lesion and postoperative chemotherapy, with other treatments including chemoembolization, radiofrequency ablation, immunologic therapy, and targeted gene therapy [5, 6]. Although the use of multiple chemotherapeutics has increased the 5-year survival rate of patients to 60–70%, the 5-year survival of osteosarcoma patients with metastasis or recurrence is only 10–20% [7, 8]. Therefore, it is of practical significance to identify new drugs with selective cytotoxicity as well as study the molecular mechanism of osteosarcoma.

Resveratrol, a natural polyphenol derived from grapes, mulberries, peanuts and other plant sources, has recently attracted great attention as an important chemopreventive agent due to its anticancer property [9–11]. The anticancer properties of resveratrol include inhibition of the proliferation of a wide variety of human tumor cells, such as breast cancer, liver cancer and uterine cancer [10, 12]. This effect may be related to the capacity of resveratrol to modulate glucose uptake and lactate production [13, 14]. Moreover, resveratrol and its compounds recently underwent a phase I clinical trial as a novel drug for anticancer therapy in men with biochemically recurrent prostate cancer [15]. However, the molecular mechanism responsible for the antitumor activity of resveratrol on osteosarcoma has not been fully elucidated.

Connexin 43 (Cx43) is an important component of gap junction intercellular communication (GJIC). Cx43/GJIC plays a key role in physiological functions, such as maintaining intercellular information and energy exchange, regulating cell growth and differentiation, and maintaining tissue homeostasis [16, 17]. Loss of Cx43 is critical to tumor progression as it allows the cells to escape growth control, resulting in uncontrolled cell proliferation and abnormal differentiation [18]. At the same time, numbers of studies have reported that the vast majority of human tumors show absent expression of Cx43 [19, 20]. Qin H et al. reported that overexpression of Cx43 genes in human breast tumor cells results in suppression of tumor growth *in vivo* [21]. In addition, Coleus forskohlii Briq can inhibit the proliferation, migration and invasion of osteosarcoma in rats by up regulating the expression of Cx43 [22]. Despite extensive studies indicating Cx43 has an anticancer effect on a wide range of human cancers, its role in osteosarcoma and the underlying mechanisms are unclear.

The Wnt/β-catenin signaling pathway is an ancient and evolutionary pathway that regulates key aspects of embryonic development and adult homeostasis [23]. Recent studies show that Wnt/β-catenin is not only closely related to tumorigenesis and bone development, but also plays an important role in tumor stem cell biology [24, 25], which also makes the Wnt/β-catenin signaling pathway a hot topic in osteosarcoma research. Without Wnt ligands, cytoplasmic β-catenin undergoes phosphorylation and degradation by a destruction complex composed of GSK-3β, adenomatous polyposis coli and axin [26]. In contrast, when Wnt ligands bind to their cell surface receptors, causing inactivation of GSK-3β, unphosphorylated β-catenin accumulates in the cytoplasm and translocates to the nucleus [27, 28], where it binds to T-cell factor/lymphocyte enhancer factor (TCF/LEF) and activates transcription of Wnt target genes, such as c-myc, cyclin D1 and matrix metalloproteinase (MMPs) [26].

In this study, we evaluate the effects of resveratrol on U2-OS cells *in vitro* and investigate the underlying mechanism involved in this process. Moreover, we also try to further clarify about the role of Cx43 in osteosarcoma and its relationship to the Wnt/β-catenin pathway.

## RESULTS

### Resveratrol inhibits the proliferation and glycolysis of U2-OS cells, and knockdown of Cx43 promotes the proliferation of U2-OS cells

**Figure 1:**
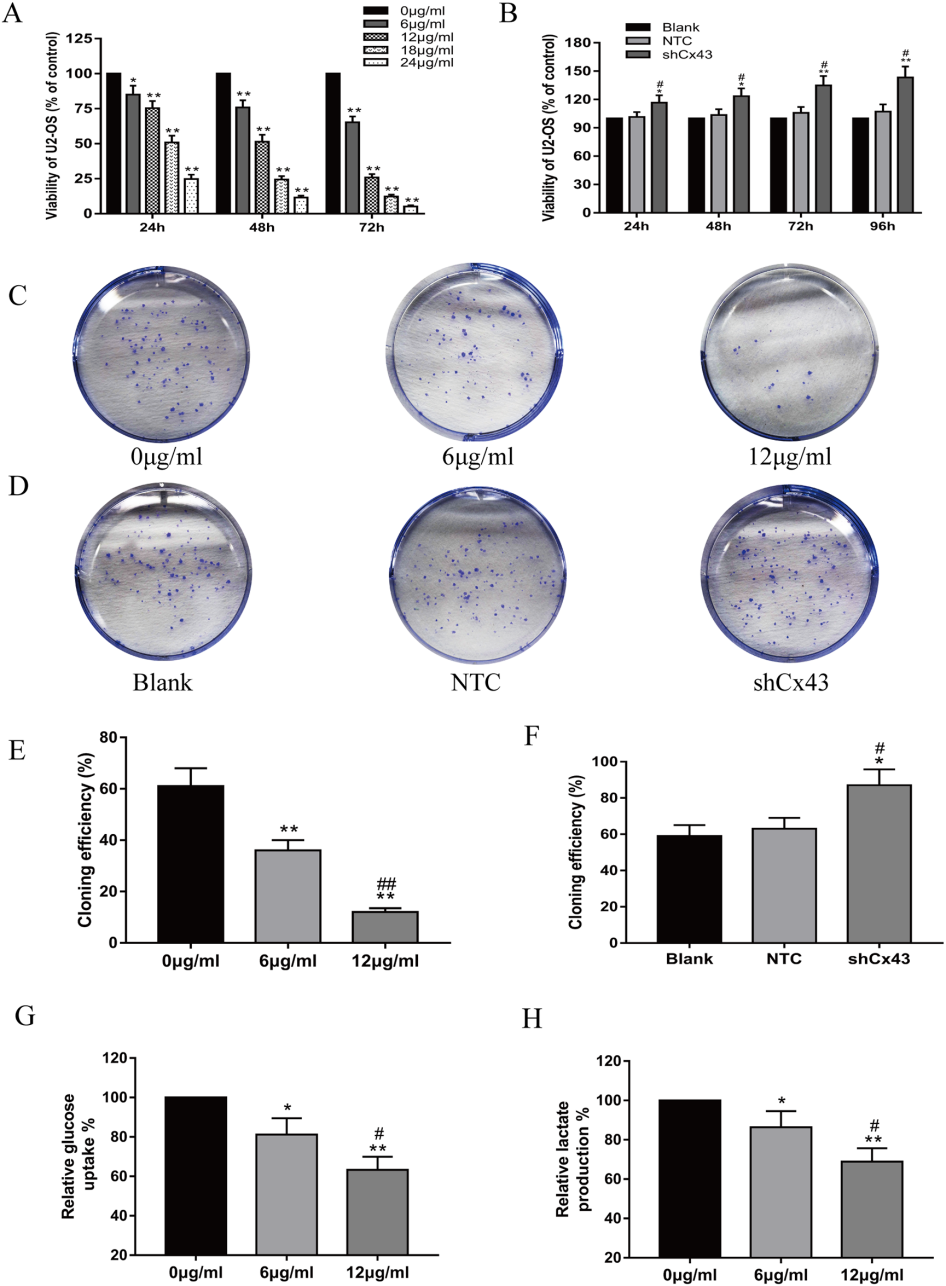
Resveratrol inhibits the proliferation and glycolysis of U2-OS cells, and knockdown of Cx43 promotes the proliferation of U2-OS cells **(A)** The inhibiting effect of resveratrol on U2-OS cell proliferation, mean ± SD, n = 3, ^*^P<0.05, ^**^P<0.01, vs. 0 μg/ml group. **(B)** Proliferation of U2-OS cells among the untreated, NTC and shCx43 groups, mean ± SD, n = 3, ^*^P<0.05, ^**^P<0.01, vs. untreated group (blank). ^#^*P*<0.05 vs. NTC group. **(C** and **D)** A macrograph of U2-OS cell colony formation following treatment with resveratrol (C) and Cx43 knockdown (D). **(E)** Cloning efficiencies of U2-OS cells following addition with resveratrol, mean ± SD, n = 3, ^**^P<0.01, vs. 0 μg/ml group. ^##^*P*<0.01 vs. 6 μg/ml group. **(F)** Cloning efficiencies of Cx43 knockdown U2-OS cells, mean ± SD, n = 3, ^*^P<0.05, vs. untreated group. ^#^*P*<0.05 vs. NTC group. **(G)** Effect of resveratrol on glucose uptake of U2-OS cells, mean ± SD, n = 3, ^*^P<0.05, ^**^P<0.01, vs. 0 μg/ml group. ^#^*P*<0.05 vs. 6 μg/ml group. **(H)** Effect of resveratrol on lactate production of U2-OS cells, mean ± SD, n = 3, ^*^P<0.05, ^**^P<0.01, vs. 0 μg/ml group. ^#^*P*<0.05 vs. 6 μg/ml group.

CCK-8 assay results showed that resveratrol inhibited U2-OS cell proliferation with a decreasing trend of concentration- and time- dependency (Figure 1A, *P*<0.05). However, the viability of cells was reduced obviously after treatment with 12 μg/ml resveratrol for 72 h, which indicated a high rate of cell death and the IC50 of resveratrol for U2-OS cell lines was found to be 12.28μg/ml after 48 h treatment. Based on this, we selected 6 μg/ml or 12 μg/ml resveratrol to treat cells for 24 h, and 12 μg/ml resveratrol to treat cells for 24 h or 48 h in the following experiments. The influence of resveratrol on colony formation by U2-OS cells was also observed (Figure 1C). The cloning efficiency of U2-OS cells was clearly decreased with increasing concentration (Figure 1E, *P*<0.01). Cx43 knockdown, NTC and blank groups were grown in 96-well plates for 24, 48, 72 and 96 h. Theproliferation of the shCx43 cells was significantly higher than either the blank or NTC group (Figure 1B, *P*<0.05). The influence of knockdown of Cx43 on the colony forming abilities of U2-OS cells was observed by performing colony formation assays (Figure 1D). Similar to the proliferation results, the cloning efficiency of Cx43 knockdown cells was significantly higher than either untreated or scrambled shRNA-expressing cells (Figure 1F, *P*<0.05). Furthermore, resveratrol inhibits glucose uptake and lactate production in U2-OS cells (Figure 1 G/H, *P*<0.05).

### Resveratrol alteres the morphology of U2-OS cells and induces apoptosis, and knockdown of Cx43 reduces apoptosis

**Figure 2:**
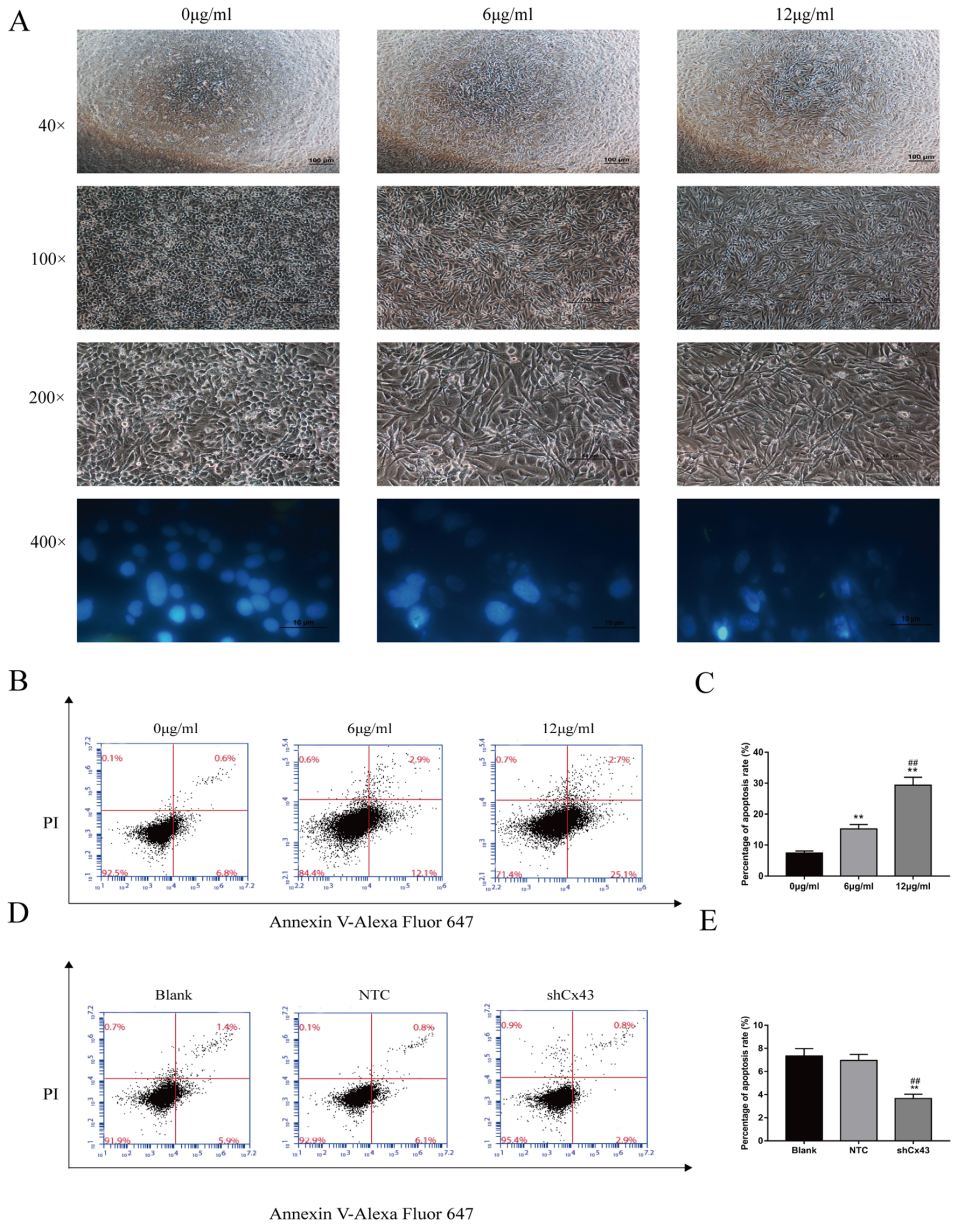
Resveratrol alters the morphology of U2-OS cells and induces apoptosis, and knockdown of Cx43 reduces apoptosis **(A)** Changes in cell morphology caused by increasing concentrations of resveratrol. **(B** and **C)** Analysis of apoptosis of U2-OS cells by flow cytometry, mean ± SD, n = 3, ^**^P<0.01, vs. 0 μg/ml group. ^##^*P*<0.01 vs. 6 μg/ml group. **(D** and **E)** Analysis of apoptosis of Cx43 knockdown U2-OS cells, mean ± SD, n = 3, ^**^P<0.01, vs. untreated group. ^##^*P*<0.01 vs. NTC group.

Following treatment with 6 μg/ml and 12 μg/ml resveratrol for 24 h, U2-OS cells were observed by bright field microscopy at 40×, 100× and 200×. The number of cells decreased gradually, and the morphology of U2OS cells changed from an oval to spindle shape. DAPI staining is a classic method to reflect the morphological changes of the cell nucleus during apoptosis. At high magnification (400×), we observed that cells presented typical apoptotic morphological changes, which included chromatic agglutination, karyopyknosis, and nuclear fragmentation (Figure 2A). Next, we detected apoptosis by flow cytometryfollowing Alexa Fluor647-conjugated Annexin V and PI double staining. After treatment with 6 μg/ml and 12 μg/ml resveratrol for 24 h, apoptosis increased with increasing concentration (Figure 2B and 2C, *P*<0.01). Knockdown of Cx43 decreased cell apoptosis compared with the untreated and NTC groups (Figure 2D and 2E, *P*<0.01).

### Resveratrol suppresses the migration and invasion of U2-OS cells, and knockdown of Cx43 enhances the migration and invasion of U2-OS cells

**Figure 3:**
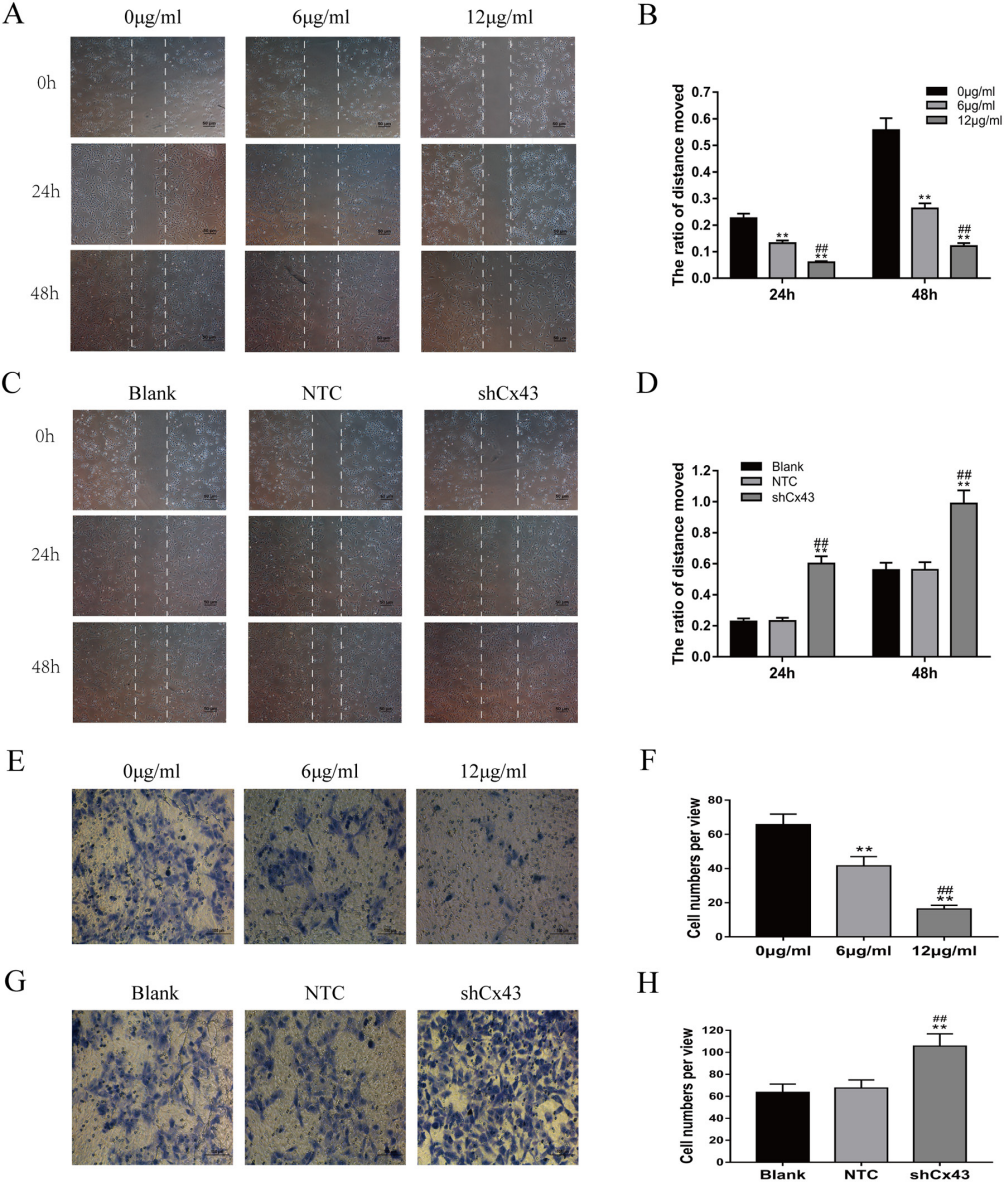
Resveratrol suppresses the migration and invasion of U2-OS cells, and knockdown of Cx43 enhances the migration and invasion of U2-OS cells **(A** and **B)** Changes of U2-OS cell migration following treatment with resveratrol, mean ± SD, n = 3, ^**^P<0.01, vs. 0 μg/ml group. ^##^*P*<0.01 vs. 6 μg/ml group. **(C** and **D)** The changes of Cx43 knockdown U2-OS cell migration, mean ± SD, n = 3, ^**^P<0.01, vs. untreated group. ^##^*P*<0.01 vs. NTC group. **(E** and **F)** Changes of U2-OS cell invasion following treatment with resveratrol, mean ± SD, n = 3, ^**^P<0.01, vs. 0 μg/ml group. ^##^*P*<0.01 vs. 6 μg/ml group. **(G** and **H)** Changes of Cx43 knockdown U2-OS cell invasion, mean ± SD, n = 3, ^**^P<0.01, vs. untreated group. ^##^*P*<0.01 vs. NTC group.

Cells were treated with 0, 6 and 12 μg/ml resveratrol for 24 and 48 h. With increased treatment time and concentration, the migration of U2-OS cells was suppressed (Figure 3A and 3B, *P*<0.01). Consistent with the scratch test, the transwell invasion assay indicated that the number of U2-OS cells that invaded from the Matrigel into the substratum of the membrane was obviously decreased after treatment (Figure 3E and 3F, *P*<0.01). Knockdown of Cx43 enhanced the migration of cells compared with the untreated and NTC groups (Figure 3C and 3D, *P*<0.01). Consistent with the scratch test, the transwell invasion assay also indicated that the invasion of cells expressing shCx43 was higher than untreated and NTC cells (Figure 3G and 3H, *P*<0.01).

### Resveratrol suppresses activity and downstream gene expression of the Wnt/β-catenin signaling pathway, and enhances Cx43 and E-cadherin gene expression

**Figure 4:**
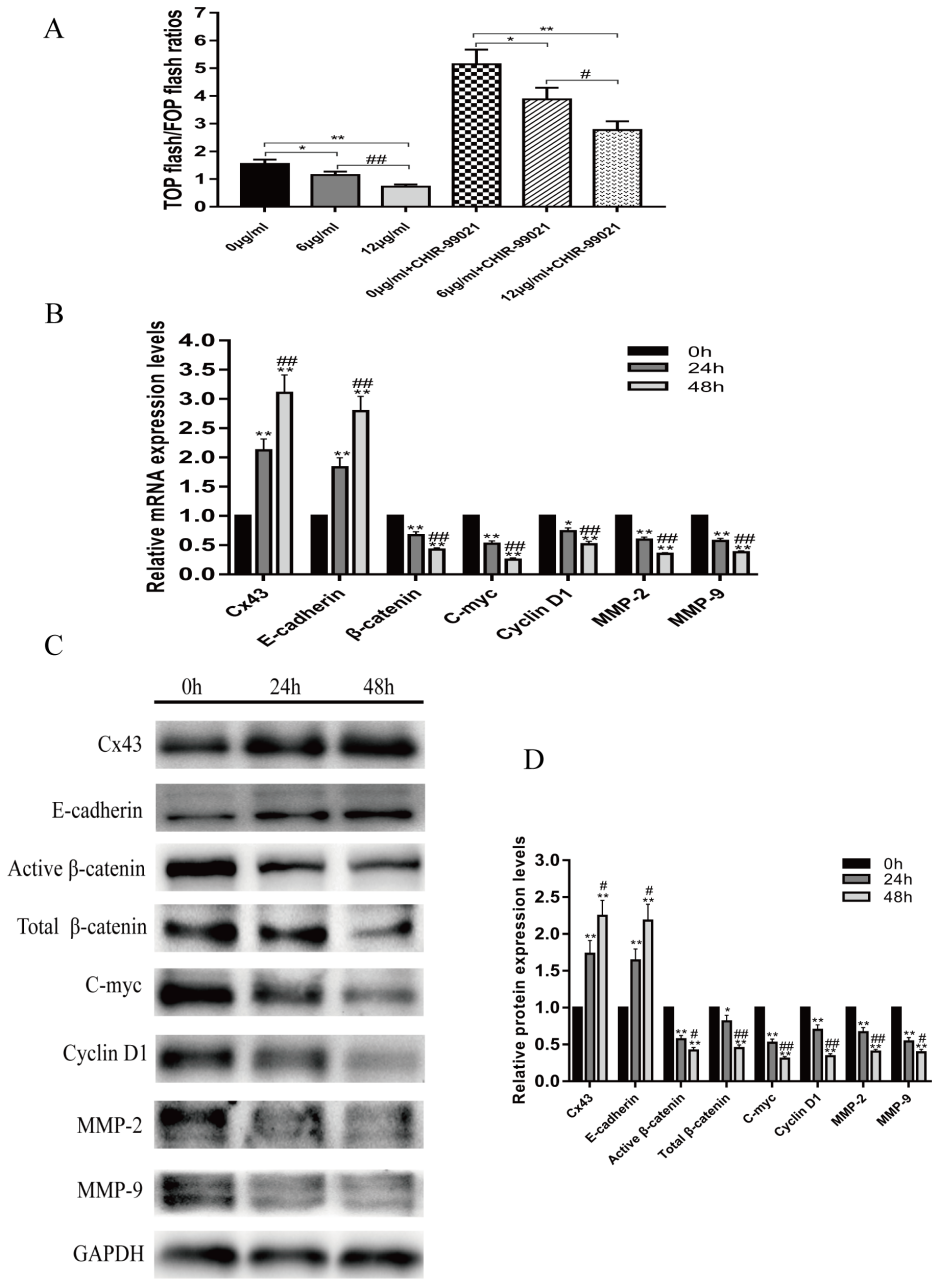
Resveratrol suppresses the Wnt/β-catenin signaling pathway **(A)** With or without pretreatment with CHIR-99021, the changes in TOP/FOP flash ratios of U2-OS cells following treatment with 0, 6 or 12 μg/ml resveratrol for 24 h, without pretreatment with CHIR-99021, mean ± SD, n = 3, ^*^P<0.05, ^**^P<0.01, vs. 0 μg/ml group. ^##^*P*<0.01 vs. 6 μg/ml group; pretreatment with CHIR-99021, mean ± SD, n = 3, ^*^P<0.05, ^**^P<0.01, vs. 0 μg/ml + CHIR-99021 group. ^#^*P*<0.05 vs. 6 μg/ml + CHIR-99021 group. **(B)** Changes in mRNA expression of Cx43, E-cadherin, β-catenin, c-myc, cyclin D1, MMP-2 and MMP-9 after treatment with 12 μg/ml resveratrol for 24 and 48 h, ^*^P<0.05, ^**^P<0.01, vs. 0 h group. ^##^*P*<0.01 vs. 24 h group. **(C** and **D)** Changes in protein expression of Cx43, E-cadherin, β-catenin, c-myc, cyclin D1, MMP-2 and MMP-9 after treatment with 12 μg/ml resveratrol for 24 and 48 h, ^*^P<0.05, ^**^P<0.01, vs. 0 h group. ^#^*P*<0.05, ^##^*P*<0.01 vs. 24 h group.

Previous studies have reported that abnormal activation of the Wnt/β-catenin signaling pathway plays an important role in osteosarcoma pathogenesis. We investigated whether resveratrol had an effect on the Wnt/β-catenin signaling pathway by using the TOP/FOP flash dual-luciferase reporter gene assay in U2-OS cells. Figure 4A shows that without CHIR-99021, an inhibitor of GSK-3β, resveratrol was able to suppress Wnt/β-catenin signaling by down-regulating the TOP/FOP flash ratio in a concentration-dependent manner (*P*<0.05). In addition, after pretreatment with CHIR-99021, the TOP/FOP flash ratio first increased, but then declined in a concentration-dependent manner after resveratrol treatment (*P*<0.05). To further study the underlying mechanism by which resveratrol inhibits U2-OS cells, we characterized the mRNA and protein expression changes of Cx43, E-cadherin, and downstream target genes of the Wnt/β-catenin signaling pathway, which included β-catenin, c-myc, cyclin D1, MMP-2 and MMP-9, using quantitative real-time polymerase chain reaction (QRT-PCR) and western blot assays. The results showed that with increasing treatment time of resveratrol, the expression levels of mRNA and protein for Cx43 and E-cadherin increased, but the mRNA and protein expression of β-catenin, c-myc, cyclin D1, MMP-2 and MMP-9 decreased (Figure 4B, 4C and 4D, *P*<0.05).

### Knockdown of Cx43 enhances the activity of the Wnt/β-catenin pathway, promotes downstream gene expression of the Wnt/β-catenin signaling pathway, and inhibits E-cadherin gene expression

**Figure 5:**
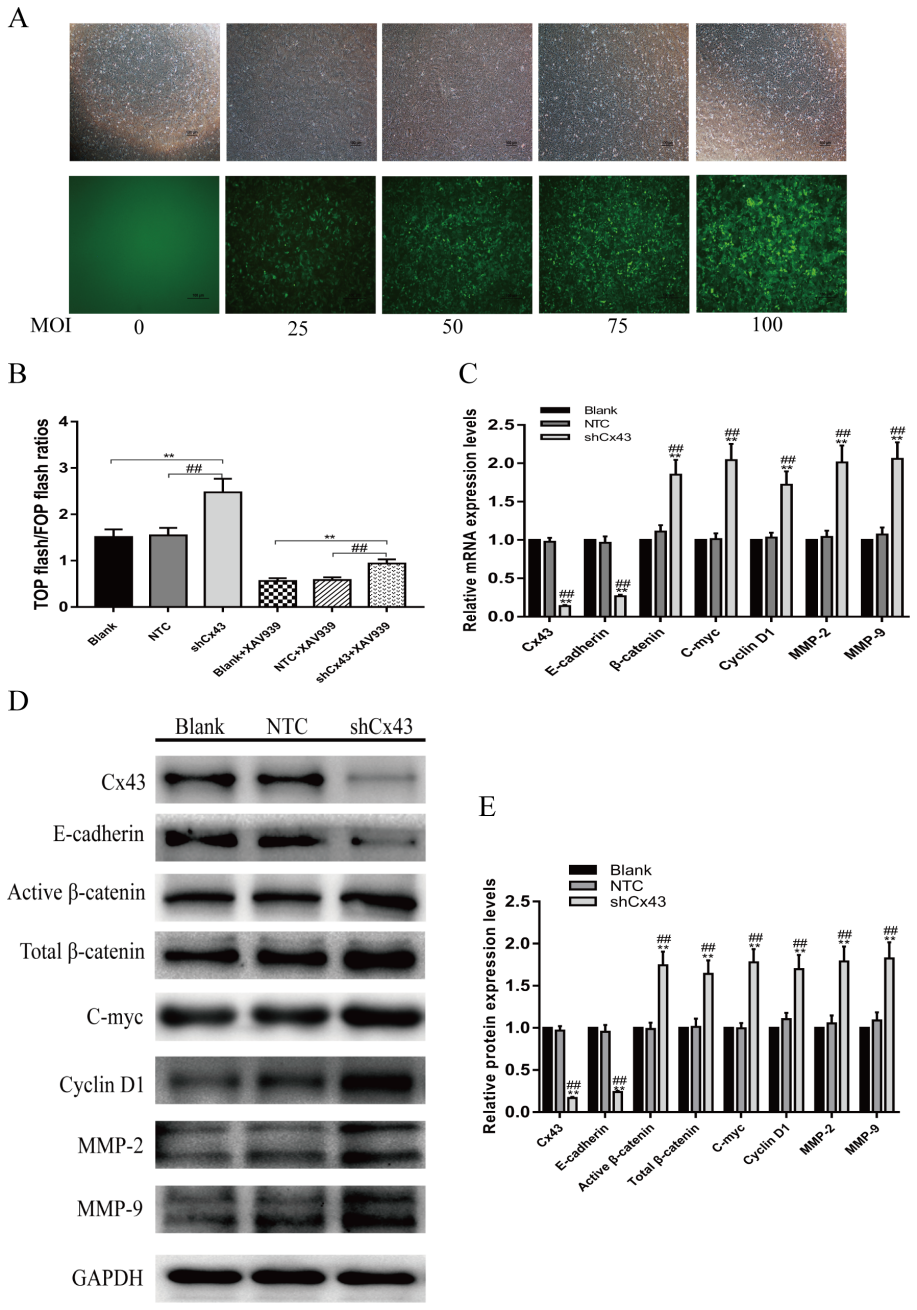
Knockdown of Cx43 enhances Wnt/β-catenin pathway activity **(A)** Fluorescence pictures of virus-infected U2-OS cells at different MOIs. **(B)** With or without treatment with XAV939, the changes of TOP/FOP flash ratios of Cx43 knockdown U2-OS cells, without treatment with XAV939, mean ± SD, n = 3,^**^P<0.01, vs. untreated group. ^##^*P*<0.01 vs. NTC group; treatment with XAV939, mean ± SD, n = 3, ^**^P<0.01, vs. untreated group + XAV939. ^##^*P*<0.01 vs. NTC group + XAV939. **(C)** Changes in mRNA expression of Cx43, E-cadherin, β-catenin, c-myc, cyclin D1, MMP-2 and MMP-9 in Cx43 knockdown U2-OS cells, mean ± SD, n = 3,^**^P<0.01, vs. untreated group. ^##^*P*<0.01 vs. NTC group. **(D** and **E)** Changes in protein expression of Cx43, E-cadherin, β-catenin, c-myc, cyclin D1, MMP-2 and MMP-9 in Cx43 knockdown U2-OS cells, mean ± SD, n = 3,^**^P<0.01, vs. untreated group. ^##^*P*<0.01 vs. NTC group.

The above results prompted us to evaluate the biological role of Cx43 in U2-OS cells. In order to further investigate the role of Cx43 in osteosarcoma progression and its relationship to the Wnt/β-catenin signaling pathway, we established Cx43 knockdown U2-OS cells by infection with lentiviruses expressing Cx43 shRNA. Viral efficacy was tested using MOIs of 0, 25, 50, 75 and 100 to infect U2-OS cells. The virus had no effect on cell viability and approximately 90% of U2-OS cells were GFP-positive at 72 hours after lentivirus transduction at 100 MOI (Figure 5A). Therefore, for knockdown studies, lentivirus transduction was carried out at 100 MOI, and cell lines stably expressing Cx43 shRNA were selected by culture in 1 μg/ml puromycin for 7 days. Figure 5C, 5D and 5E show that the mRNA and protein expression levels of Cx43 were efficiently knocked down in U2-OS cells compared with untreated and scrambled shRNA-expressing cell (*P*<0.01). Figure 5B shows that without XAV939, an inhibitor of β-catenin, knockdown of Cx43 was able to activate the activities of Wnt/β-catenin signaling, as judged by the up-regulation the TOP/FOP flash ratio (*P*<0.01). In addition, after treatment with XAV939, the TOP/FOP flash ratio was dropping overall, but the TOP/FOP flash ratio in the Cx43 shRNA-expressing group was still significantly higher than that in the untreated and NTC groups *P*<0.01). To investigate whether the expression of Cx43 is also associated with Wnt/β-catenin signaling, we detected the mRNA and protein expression changes of E-cadherin, and the downstream target genes of Wnt/β-catenin signaling pathway, including β-catenin, c-myc, cyclin D1, MMP-2 and MMP-9, using QRT-PCR and western blot assays. After knockdown of Cx43, the expression levels of mRNA and protein of E-cadherin genes decreased, but the mRNA and protein expression of β-catenin, c-myc, cyclin D1, MMP-2 and MMP-9 were up-regulated compared with untreated and NTC cells (Figure 5C, 5D and 5E, *P*<0.05). These results suggest that Cx43 expression is negatively related to the activity of the Wnt/β-catenin pathway in U2-OS cells.

## DISCUSSION

Osteosarcoma is the most common clinical primary malignant bone tumor and is characterized by the proliferation of neoplastic cells, producing immature bone or osteoid tissue, with a high propensity for neovascularization and high-risk distant metastasis [29]. Resveratrol is a natural polyphenols derived from a variety of plants, which affects all stages of carcinogenesis (initiation, promotion, and progression) by modulating a variety of critical signaling pathways [30, 31]. Ko YC et al. [32] reported that resveratrol induced differentiation and apoptosis in anaplastic large-cell lymphoma cells by enhance the expression of death receptor Fas/CD95. Benitez DA et al. [33] also indicated that the regulation of prostate cancer cells survival by resveratrol involves inhibition of nuclear transcription factor-κB (NF-κB). The study of Yang YT et al. [34] demonstrated that resveratrol inhibits invasion of human lung adenocarcinoma cells by suppressing the MAPK pathway. In this study, we explored the effect of resveratrol on osteosarcoma cells and investigating the related mechanisms involved in this process.

We show that resveratrol treatment reduces the proliferation of cells in a time- and concentration-dependent manner, and decreases the cell cloning efficiency. Moreover, our data indicated that the glucose uptake and lactate production were inhibited by resveratrol treatment in U2-OS cells, which was consistent with the previous study that resveratrol inhibited glycolysis in other cancers [13, 35]. At the same time, we observed that the morphology of U2-OS cells changes from an oval to spindle shape, similar to bone mesenchymal stem cells, after treatment with resveratrol. DAPI staining showed that resveratrol treatment led to karyopyknosis, chromatic agglutination and nuclear fragmentation, which are typical apoptotic morphological changes [36]. Flow cytometry analysis also indicated that the apoptosis of U2-OS cells is increased by resveratrol treatment. Cell migration is the basis of tissue development and underlies pathological conditions, such as tumor invasions, the key process of cancer metastasis [37]. The effects of resveratrol on migration and invasion of U2-OS cells were assessed through a scratch test and transwell invasion assay. The results showed that the migration of U2-OS cells is inhibited and the number of cells that move from the Matrigel into the substratum of the membrane is decreased by resveratrol application. Therefore, we conclude that resveratrol suppresses the proliferation, glycolysis, migration and invasion of U2-OS cells and induces apoptosis.

Previous studies have reported that the Wnt/β-catenin signaling pathway is widely expressed in bone tissue and cells, and its aberrant activation is closely associated with the progression of osteosarcoma [25, 38]. In this study, we explored the effect of resveratrol on the Wnt/β-catenin signaling pathway by using the TOP/FOP flash plasmid dual-luciferase reporter assay, which is a classical method to evaluate the transcriptional activity of TCF/LEF in Wnt/β-catenin signaling [39]. The TOP/FOP flash ratio dramatically decreased in a resveratrol concentration-dependent manner, demonstrating that the transcriptional activity of TCF/LEF is suppressed by resveratrol. We also show that even when Wnt/β-catenin signaling is pre-activated by CHIR-99021, the TOP/FOP flash ratio is still reduced by resveratrol treatment. Therefore, we speculated that resveratrol may inhibit the activity of the Wnt/β-catenin signaling pathway and the expression of its related genes, such as E-cadherin, β-catenin, c-myc, cyclin D1, MMP-2 and MMP-9.

E-cadherin is a transmembrane glycoprotein that regulates cell recognition, migration, differentiation and the formation of tissues and organs, and the complex formed by E-cadherin and β-catenin is the molecular basis for maintenance of normal polarity and intercellular adhesion by epithelial cells [40]. A decrease of E-cadherin expression is closely related to tumor development, invasion and metastasis [41], and studies have shown that E-cadherin inhibits the Wnt/β-catenin pathway through its binding at the C-terminal domain of β-catenin at the plasma membrane, thereby sequestering β-catenin from the cytoplasmic pool [42]. β-Catenin is a crucial signaling molecule in the Wnt/β-catenin signaling pathway, and dephosphorylation of β-catenin at Ser33, Ser37, and Thr41 activates β-catenin [43]. C-myc is an important member of the proto-oncogene family that regulates cell proliferation, differentiation, and programmed cell death [44]. Its overexpression is one of the most common changes in the development of human cancers, and studies also show that the suppression of the c-myc oncogene induces cellular senescence in diverse tumor types, including osteosarcoma [45]. Cyclin D1 is a key cell-cycle regulator, and its overexpression can shorten the cell cycle and lead to abnormal cell proliferation, inducing a variety of tumors, including osteosarcoma [46, 47]. Matrix metalloproteinases (MMPs) can degrade almost all the extracellular matrix components, and play a vital role in tumor invasion and metastasis [48, 49]. In osteosarcoma patients, MMP-2 and MMP-9 are generally overexpressed [50].

The mRNA and protein expression of genes related to the Wnt/β-catenin signaling pathway were detected by QRT-PCR and western blot. As expected, both assays indicated that with the prolonging of treatment time of resveratrol, the mRNA and protein expression of E-cadherin was increased, but the mRNA and protein expression of β-catenin, c-myc, cyclin D1, MMP-2 and MMP-9 were down-regulated. Additionally, we also found that the levels of active and total β-catenin were decreased in response to varying treatment times. These results further demonstrate that resveratrol suppresses the activity of the Wnt/β-catenin signaling pathway.

Cx43 is one of the vital gap junction proteins. Currently, the role of Cx43 as a tumor suppressor has been supported by most studies [19, 20]. In recent years, the absence of of Cx43 expression has been observed in many types of cancers, including breast cancer, colorectal cancer and esophageal cancer [19, 20, 51], suggesting that Cx43 may play an important role in tumorigenesis. In our QRT-PCR and Western blot experiments, we showed that the activity of the Wnt/β-catenin signaling pathway decreases after resveratrol treatment while the mRNA and protein expression of Cx43 is significantly increased, suggesting that Cx43 may be negatively related to the activity of the Wnt/β-catenin pathway in U2-OS cells. However, the role of Cx43 in osteosarcoma and its relationship with Wnt/β-catenin pathway remains unclear.

Subsequently, through shRNA knockdown of Cx43, we find that the mRNA and protein expression of E-cadherin is decreased, but the mRNA and protein expression of β-catenin, c-myc, cyclin D1, MMP-2 and MMP-9 are upregulated. In addition, we also find that the levels of active and total β-catenin are increased. Moreover, the dual-luciferase assay results show that the TOP/FOP flash ratio increases whereas knockdown of Cx43, even after inhibition of β-catenin by XAV939, the TOP/FOP flash ratio was dropping overall, but the TOP/FOP flash ratio in the Cx43 knockdown group is still significantly higher than that in untreated and scrambled shRNA-expressing groups. These results indicate that knockdown of Cx43 can activate the Wnt/β-catenin signaling pathway, and Cx43 may negatively regulate Wnt/β-catenin signaling pathway in U2-OS cells. Furthermore, our results also demonstrate that knockdown of Cx43 enhances the proliferation, migration and invasion of U2-OS cells, and reduces apoptosis.

In summary, we demonstrate that the antitumor activity of resveratrol against osteosarcoma U2-OS cells *in vitro* is achieved by up-regulating Cx43 and E-cadherin expression, and suppressing the Wnt/β-catenin signaling pathway. Moreover, knockdown of Cx43 can activate the Wnt/β-catenin signaling pathway, suggesting that Cx43 expression is negatively related to the activity of the Wnt/β-catenin pathway in U2-OS cells.

## MATERIALS AND METHODS

### Cell culture

Human osteosarcoma U2-OS cell lines, derived from the Cell bank of Chinese Academy of Sciences (Shanghai, China), were cultured in RPMI 1640 medium (Gibco, NY, USA) containing 10% (v/v) fetal bovine serum (FBS, Gibco, NY, USA), and 1% (v/v) penicillin-streptomycin solution (Hyclone, UT, USA). Cells were cultured in a humidified incubator, containing 5% CO_2_, at 37°C. Resveratrol was purchased from Sigma-Aldrich Chemical Co. (CA, USA), and the purity was approximately 99%, as analyzed by HPLC. Resveratrol was dissolved in DMSO (Sigma, CA, USA) and diluted with medium. As a vehicle control, cultured cells were incubated in medium containing DMSO at a final concentration of less than 0.1%.

### Lentivirus infection

Lentiviral vectors (H1/GFP&Puro) carrying CX43 short-hairpin RNAs (Cx43 shRNAs), termed sh1 (5‘- GAACCTACATCATCAGTAT -3’), sh2 (5‘- GGCTAATTACAGTGCAGAA -3’) and sh3 (5‘- CAGTCTGCCTTTCGTTGTA-3’), were constructed by GenePharma (Suzhou, China) to knockdown CX43 expression. A vector containing scrambled shRNA (5‘-CAACAAGATGAAGAGCACCAA -3’), termed NTC (a negative control), was also constructed. The virus titer used for infection was 10^9^ TU/ml. U2-OS cells were inoculated into 6-well plates at a density of 4×10^5^ per well and allowed to attach overnight. Then cells were cultured with medium containing 5 μg/ml polybrene (Sigma, CA, USA) and incubated for one hour prior to addition of lentivirus at a multiplicity of infection (MOI) between 25 to 100. The incubation medium was replaced with fresh medium 24 hours after infection, and then cells were screened with medium containing 1 μg/ml puromycin (Sigma, CA, USA) for 7 days to construct stable expression cell lines for functional analysis. The fluorescence of GFP was detectable using an inverted fluorescence microscope (Nikon, Tokyo, Japan). Our preliminary experimental results showed that sh1 and sh2 significantly knocked down CX43 (88% and 62%, respectively), while sh3 had no obvious efficiency. No knockdown was observed in NTC group. Therefore, we chose sh1 as target sequence for the formal experiment.

### Cell counting kit-8 proliferation assay

For the resveratrol group, U2-OS cells were inoculated in a 96-well dish at a final density of 5×10^3^ cells/well and incubated for 24 h. Then cells were treated with resveratrol at varying concentrations (0, 6, 12, 18, 24 μg/ml) for 24, 48 and 72 h. For lentivirus-transduced cells, cell lines stably expressing Cx43 shRNA (shCx43), scrambled shRNA (NTC), and normal U2-OS cells (blank), were grown in 96-well plates for 24, 48, 72 and 96 h (initially planted at 5×10^3^ cells/well). Thereafter, cell viability was determined by a Cell Counting Kit-8 (Dojindo, Kumamoto, Japan) according to the manufacturer’s instructions, and absorption at 450 nm was determined for each sample using a 96-well plate microplate reader (Thermo Scientific, MA, USA). Cell viability (%) = [OD (treated)-OD (blank)]/[OD (control)-OD (blank)] ×100%.

### Colony formation assay

For the resveratrol group, 100 U2-OS cells were inoculated into 6-well plates and incubated overnight, then the cells were treated with 0, 6 or 12 μg/ml resveratrol and cultured for 12 days, during which the medium containing the corresponding concentrations of resveratrol was refreshed every 2 days. For the lentivirus-infected groups, 100 cells of shCx43, NTC and blank groups were plated in a 6-well plate and maintained in medium containing 10% FBS for 12 days. Thereafter, the colonies were fixed with 4% paraformaldehyde for 20 min and stained with 0.5% crystal violet for 15 min. Colonies with than 50 cells were scored, and the number of clones was counted in triplicate wells.

### Glucose uptake and lactate production assays

U2-OS cells were seeded in 10 cm plates and incubated overnight, then the cells were treated with 0, 6 or 12 μg/ml resveratrol and cultured for 24 h. Cells were trypsinized and seed in 6-well plates (4×10^5^ cells/well) with fresh medium for 12 h. Glucose and lactate levels were measured in medium using the Automatic Biochemical Analyzer 7180 (HITACHI, Tokyo, Japan). The results were normalized by the protein amounts of each sample.

### DAPI staining

U2-OS cells were inoculated in 24-well plates and treated with 0, 6 or 12 μg/ml resveratrol for 24 h, then cells were rinsed three times with phosphate-buffered saline (PBS), and 0.2% Triton X-100 was added to permeabilize the cell membrane for 15 min, and DAPI (Beyotime, Shanghai, China) was added to stain nuclei for 15 min in the dark. Cell nuclei were observed and photographed using an inverted fluorescence microscope.

### Apoptosis analysis by flow cytometry

For the resveratrol group, U2-OS cells were inoculated into 12-well plates overnight, then exposed to 0, 6 or 12 μg/ml resveratrol for 24 h. Subsequently, the cells were collected and resuspended in 400 μl of 1× binding buffer. Then 100 μl cell suspension was placed in a 5 ml flow tube, 5 μl Annexin V/Alexa Fluor647 (Beijing 4A Biotech Co., Beijing, China) was added, and the mixture was incubated at room temperature for 5 min in the dark. Thereafter, 10 μl propidium iodide (PI) and 400 μl PBS were added. The fluorescence intensity of the cells was analyzed immediately using a BD AccuriTM C6 (BD, NJ, USA) flow cytometer. For lentivirus-infected cells, stably expressing cell lines (shCx43, NTC) and blank groups were assessed for cell apoptosis analysis as described above.

### Wound healing scratch test

For Resveratrol group, U2-OS cells were inoculated into a 6-well plate and incubated for 24 h. A 200 μl of pipette tip was used for scratching, then the medium was discarded and cells were rinsed twice by PBS. Subsequently, cells were treated with 0, 6 and 12 μg/ml resveratrol for 24 and 48 h. Resveratrol was diluted with medium containing 2% FBS to eliminate the possible influence of cell media on proliferation. The migration of cells in each group was observed and photographed under an inverted fluorescence microscope. Image J software was used to measure scratch areas, and the migration of osteosarcoma cells was expressed as the ratio of the distance moved (= [D (different time points) - D (0 h)] / D (0 h)).

### Transwell invasion assay

For the resveratrol group, U2-OS cells were starved in serum-free medium for 24 h and then resuspended in serum-free medium containing 0, 6 or 12 μg/ml resveratrol, and the cell density adjusted to 3 × 10^5^ cells/ml. Then, 100 μl cell suspension was added into the upper chamber of a transwell pre-coated with Matrigel (BD, NJ, USA), while the lower chamber was filled with 600 μl of medium containing 20% FBS as the chemo-attractant. After incubation for 24 h, the non-invaded cells of the upper chamber were carefully removed with sterile swabs, and invaded cells were fixed with 4% paraformaldehyde for 20 min and stained with 0.5% crystal violet for 15 min, then rinsed thrice with PBS. Using an inverted microscope, 5 fields were randomly selected for each group, and the numbers of cells went through Matrigel were counted under high magnification (200×).

### Dual luciferase assay

U2-OS Cells were inoculated in a 96-well (5×10^3^ cells/well) and were incubated for 24 h, and then transfected with 200 ng TOP-Flash/FOP-Flash (Millipore, MA, USA), and 20 ng pRL-TK vector expressing Renilla luciferase (Promega, WI USA) following the recommended protocol using Lipofectamine 3000 (Thermo Scientific, MA, USA) for another 24 h. Subsequently, the assays were divided into four groups: resveratrol (0, 6 or 12 μg/ml resveratrol for 24 h), CHIR-99021 (inhibitor of GSK-3α/β, Selleck, TX, USA) + resveratrol (the cells were pretreated with 10 μm CHIR-99021 for 24 h to activate the Wnt/β-catenin signaling pathway); lentivirus infection (shCx43, NTC and Blank), and lentivirus infection + XAV939 [(inhibitor of β-catenin, Selleck, TX, USA), the cells were treated with 10 μm XAV939 for 24 h to suppress the Wnt/β-catenin signaling pathway]. The OD values of the TOP flash and the FOP flash were detected by a dual-luciferase reporter assay system (Promega, WI, USA) from cell lysates, and the TOP/FOP ratio reflected the activity of the Wnt/β-catenin signaling pathway.

### Quantitative real-time polymerase chain reaction (QRT-PCR)

Total RNA was extracted with an RNA extraction kit (TaKaRa, Shiga, Japan) according to the manufacturer’s instructions, and total RNA concentration was determined with a SmartSpec Plus spectrophotometer (BIO-RAD, CA, USA). First-strand cDNA was synthesized using PrimeScript™ RT Master Mix (TaKaRa, Shiga, Japan) from 1000 ng total RNA in a 20 μl total reaction volume. QRT-PCR was performed with SYBR® Premix Ex Taq™ (TaKaRa, Shiga, Japan) using a CFX96 QPCR Detection System (BIO-RAD, CA, USA) in a total reaction volume of 25 μL. Primer sequences are listed in Table 1. Glyceraldehyde-3-phosphate dehydrogenase (GAPDH) was used as the internal control, and relative gene expression levels were calculated by means of the 2^-ΔΔCt^ method. Each experiment was performed at least three times independently.

**Table 1:** PCR primer sequences for quantitative real-time polymerase chain reaction

Gene	Forward primer sequence	Reverse primer sequence
Cx43	GGGTGACTGGAGCGCCTTAG	TTATCTCAATCTGCTTCAAG
E-cadherin	GCCGCTGGCGTCTGTAGGAA	TGACCACCGCTCTCCTCCGA
β-catenin	AAAATGGCAGTGCGTTTAG	TTTGAAGGCAGTCTGTCGTA
C-myc	CCACACATCAGCACAACTACG	CCGCAACAAGTCCTCTTCAG
Cyclin D1	TCGGGAGAGGATTAGGTTCC	GTCACTGGATGGTTTGTTGG
MMP-2	GACCACAGCCAACTACGATG	CACAGTCCGCCAAATGAAC
MMP-9	CATCGTCATCCAGTTTGGTGT	AGGGTTTCCCATCAGCATT
GAPDH	CCCTTCATTGACCTCAACTAC	CCACCTTCTTGATGTCATCAT

### Western blot analysis

Total protein was extracted with RIPA lysis buffer (Beyotime, Shanghai, China) containing 1% (v/v) protease inhibitor cocktail (Bimake, TX, USA) according to the manufacturer’s protocol. The protein concentration was detected using a BCA Kit (Beyotime, Shanghai, China). All the samples were mixed with 5×sodium dodecyl sulfate (SDS) loading buffer (1:4), boiled for 10 min and stored at −80°C for later use. Equal amounts of protein were separated on SDS-PAGE gels (8–10% separation gel) and transferred onto polyvinylidene fluoride membranes (PVDF, Millipore, MA, USA). After blocking in 5% nonfat milk for 1 h at room temperature, membranes were incubated with corresponding rabbit primary antibodies overnight at 4°C. Antibodies included rabbit anti-Cx43, rabbit anti-E-cadherin, rabbit anti-β-catenin, rabbit anti-active β-catenin, rabbit anti-c-myc, rabbit anti-cyclin D1, rabbit anti-MMP-2, rabbit anti-MMP-9 (1:1000, Cell Signaling Technology, MA, USA), and rabbit anti-GAPDH (1:1000, Panera, GuangZhou, China). GAPDH was used as the internal control for the total protein. Secondary antibody used was horseradish peroxidase (HRP)-conjugated goat anti-rabbit IgG (1:10000, Jackson, PA, USA). Protein bands were visualized using an enhanced chemiluminescence reagent (Thermo Scientific, MA, USA), imaged with Bio-Rad ChemiDoc-XRS+ (BIO-RAD, CA, USA), and analyzed using Image Lab software (BIO-RAD, CA, USA).

### Statistical analysis

Data are presented as the means ± standard deviation (SD) of three independent experiments, and were analyzed with SPSS 19.0 software (SPSS Inc., IL, USA). Statistical analysis was calculated using Student’s two-tailed t-test, one-way or two-way analysis of variance (ANOVA). A *p*-value <0.05 was considered statistically significant.
